# Polyphenolic HRMS Characterization, Contents and Antioxidant Activity of *Curcuma longa* Rhizomes from Costa Rica

**DOI:** 10.3390/antiox11040620

**Published:** 2022-03-24

**Authors:** María Isabel Quirós-Fallas, Felipe Vargas-Huertas, Silvia Quesada-Mora, Gabriela Azofeifa-Cordero, Krissia Wilhelm-Romero, Felipe Vásquez-Castro, Diego Alvarado-Corella, Andrés Sánchez-Kopper, Mirtha Navarro-Hoyos

**Affiliations:** 1 Bioactivity & Sustainable Development (BIODESS) Group, Department of Chemistry, University of Costa Rica (UCR), San Jose 2060, Costa Rica; maria.quirosfallas@ucr.ac.cr (M.I.Q.-F.); luis.vargashuertas@ucr.ac.cr (F.V.-H.); krissia.wilhelm@ucr.ac.cr (K.W.-R.); manuel.vasquezcastro@ucr.ac.cr (F.V.-C.); luis.alvaradocorella@ucr.ac.cr (D.A.-C.); 2 Department of Biochemistry, School of Medicine, University of Costa Rica (UCR), San Jose 2060, Costa Rica; silvia.quesada@ucr.ac.cr (S.Q.-M.); gabriela.azofeifacordero@ucr.ac.cr (G.A.-C.); 3 Centro de Investigación y de Servicios Químicos y Microbiológicos (CEQIATEC), Department of Chemistry, Costa Rica Institute of Technology (TEC), Cartago 7050, Costa Rica; ansanchez@itcr.ac.cr

**Keywords:** *Curcuma longa*, curcumin, demethoxycurcumin, bisdemethoxycurcumin, polyphenols, medicinal herbs, UPLC, QTOF-ESI MS

## Abstract

*Curcuma longa* constitutes an important source of secondary metabolites that have been associated with multiple health benefits. For instance, curcumin, demethoxycurcumin and bisdemethoxycurcumin, have been found to perform important biological activities, such as anti-inflammatory, antioxidant, anticancer, antimicrobial, antihypertensive and anticoagulant. These promising results prompted this research to evaluate the polyphenols of *C. longa* rhizomes in Costa Rica. The present work reports a comprehensive study on the polyphenolic profile and the contents of the three main curcuminoids as well as the antioxidant activity of extracts from *C. longa* rhizomes (*n* = 12) produced in Costa Rica. Through UPLC-QTOF-ESI MS, a total of 33 polyphenols were identified, grouped in eight types of structures. In addition, our findings on the main curcuminoids using UPLC-DAD show all rhizomes complying with total curcuminoids (TC) content established by the United States Pharmacopeia (USP). At an individual level, samples NW-3 and NE-1 show the higher contents (118.7 and 125.0 mg/g dry material), representing more than twice the average values of the lowest samples. These samples also exhibit the highest Folin–Ciocalteu (FC) reducing capacity results as well as the best DPPH (IC_50_ 15.21 and 16.07 µg extract/mL) and NO (IC_50_ between 52.5 and 54.3 µg extract/mL) antioxidant values. Further, Pearson correlation analysis findings indicated positive correlation (*p* < 0.05) between TC, CUR with FC results (r = 0.833 and r = 0.867 respectively) and negative correlation (*p* < 0.05) between CUR, TC and FC with DPPH results (r = −0.898, r = −0.911, and r = −0.890, respectively) and between NO results and DPPH (r = −0.805, *p* < 0.05). Finally, results for Principal Component Analysis (PCA) showed composition variability associated with their region of origin with products from the Northeastern (NE) region exhibiting higher average values for FC, TC and antioxidant activities. Further, PCA confirmed that two samples, namely NE-1 and NW-3, stand out by presenting the highest PC1 due to their particularly high TC, CUR and antioxidant activities. Consequently, our findings agree with previous results indicating the importance of *C. longa* extracts to elaborate products with potential benefits for health, while delivering extracts with higher levels of curcuminoids than previous reports and exhibiting high antioxidant activity.

## 1. Introduction

Turmeric species are found throughout the South and South East Asian countries, with a few species extending their distribution to South Pacific and Australia [1]. *Curcuma longa* in particular is the most common and due to its medicinal properties, has been domesticated in several other regions including Central and South America [2].

Among their phytochemical profile, polyphenols are the most abundant type of compound in turmeric. Numerous studies have demonstrated the role played by polyphenols as health modulators. Their versatility encompasses not only as powerful preventive substances but also as therapeutical agents. Those characteristics are specially embodied by curcuminoids, the main and most abundant category in turmeric [3]. Curcuminoids are diketone molecules, formally classified as diarylheptanoids with different functional groups that allows them to react and interact with several biochemical machinery [4].

Reactive oxygen species play an important role in control of inflammatory outcomes and are critical for an appropriate response against pathogens and diseases [5,6]. It has been reported that the antioxidant nature of curcuminoids, associated with its ability to eliminate free radicals through its reactive sites, allows them to act in the prevention of metabolic and cardiovascular diseases [7,8].

Other studies have found promising evidence for the therapeutic effects of curcuminoids in degenerative and autoimmune diseases involving inflammatory processes such as multiple sclerosis [9], psoriasis [10], osteoarthritis [11], ulcerative colitis [12], diabetes and cardiovascular diseases [13], cancer [14], abnormal pulmonary inflammatory responses [15] and in modulating the immune response to counteract the SARS-CoV-2 infections [16,17].

Antioxidant balance is key to maintain optimal health conditions and the alteration of such balance is a common factor in diseases. Studies on the contents of secondary metabolites in natural products, which can act as potential antioxidant sources, have thus become more important, which in turn accounts for increased interest in the scientific studies of foods such as turmeric [18]. 

Hence, the objective of this work was to obtain extracts of *C. longa* rhizomes produced in different parts of Costa Rica (*n* = 12), using Pressurized Liquid Extraction (PLE), which has been used to improve polyphenols extraction [19,20], in order to characterize their polyphenols through UHPLC-QTOF-ESI MS. In addition, our work aimed to determine the main curcuminoids contents using UHPLC-DAD and to evaluate their antioxidant activity through FC, DPPH and NO methods, applying correlation studies and Principal Component Analysis (PCA) to the data obtained. Further, to the best of our knowledge, this is the first detailed study on turmeric from Central America.

## 2. Materials and Methods

### 2.1. Curcuma longa Rhizomes, Chemicals and Reagents

Rhizomes from *C. longa* were acquired in ripe state from producers from different places in Costa Rica, namely four in the Northern region (NR-1, NR-2, NR-3, NR-4), four from the Northeastern region (NE-1, NE-2, NE-3, NE-4), three from the Northwestern region (NW-1, NW-2, NW-3) and one from the Western region (WR-1). Solvents of ACS or HPLC grade, for instance methanol, acetonitrile and acetone were acquired from Baker (Center Valley, PA, USA). Reagents such as curcumin standard, sodium molibdate, gallic acid, 2,2-diphenyl-1-picrylhidrazyl (DPPH), gallic acid, and sodium tungstate sodium nitroprusside (SNP), naphthylethylenediamine dihydrochloride, sodium nitrite and sulfanilamide, were obtained from Sigma-Aldrich (St. Louis, MO, USA).

### 2.2. Extraction and Quantification of Phenolic Compounds from C. longa Rhizomes

To determine the best conditions for extraction of *C. longa* rhizomes, extraction processes were carried out in a Pressurized Liquid Extraction (PLE) equipment (Accelerated Solvent Extractor, Dionex™ASE™300 Accelerated Solvent Extractor (Thermo Scientific™, Walthman, MA, USA). A factorial 2^3^ design (FD) was performed using three-factors with two-levels each for the extraction process, namely solvents (methanol and acetone), two conditions of temperature (60 and 80 °C) and two different extraction static times (6 and 10 min). The details and sequence of the FD experiments is summarized in Section 3.1 from Results and Discussion. The efficiency of the extractions was determined using and UltiMate U3000 (Thermo Scientific, Walthman, MA, USA) UPLC-DAD system for the quantification of the three main curcuminoids, namely curcumin (CUR), demethoxycurcumin (DMC) and bisdemethoxycurcumin (BDM), based in the United States Pharmacopeia (USP) chromatographic method [21]. Quantification of these compounds was performed against the calibration curve of curcumin standard (Sigma-Aldrich, St. Louis, MO, USA), using a Luna RP-C18 column (150 mm × 4.6 mm i.d. × 4 µm, Phenomenex, Torrance, CA, USA) with a pre-column filter (Phenomenex, Torrance, CA, USA) at 25 °C. Solvents used in the mobile phase were water (A), methanol (B) and acetonitrile (C), and an isocratic elution program of 45% A, 15% B and 40% C was applied. The DAD was operating at 250–420 nm. For the calibration curve of CUR, the limit of detection (LOD) and the limit of quantification (LOQ) were 0.6 and 1.9 ppm respectively, for DMC were 1.1 ppm and 3.5 ppm respectively and for BDM were 1.0 ppm and 3.5 ppm respectively. Once the optimal conditions were established, *C. longa* extracts (*n* = 12) were obtained from the corresponding dry material using a Dionex™ASE™300 Accelerated Solvent Extractor (Thermo Scientific™, Walthman, MA, USA) at a temperature of 80 °C and 10 min static time for 3 cycles and employing acetone as solvent. The extracts were dried out using a Buchi™215 (Flawil, Switzerland) rotavapor to determine the extract yield. UPLC-DAD quantification for the three main curcuminoids was carried-out according to the chromatographic method described above. 

### 2.3. UPLC-QTOF-ESI MS

The UPLC-MS system used to analyze the composition of *C. longa* extracts consisted of a Xevo G2-XS QTOF (Waters, Wilmslow, UK) coupled with an AQUITY H Class UPLC system with quaternary pump. ESI source parameters were set to a capillary voltage of 2 kV, sampling cone of 20 eV, source temperature of 150 °C, and source offset of 10 °C. The desolvation temperature was set at 450 °C, the cone gas flow at 0 L/h and the desolvation gas flow at 900 L/h.

Measurement was performed in MS^e^ high resolution negative mode using an acquisition mass range from 100 *m*/*z* to 2000 *m*/*z* and a scan rate of 0.5 s, where fragmentation was carried out using Independent Data Acquisition with a collision energy ramp from 20 V to 30 V storing at the high energy function. Instrument calibration was applied in the mass range of the measurement with sodium formate. Lock mass correction was applied directly to the measurement using leucine enkephalin infusion measured each 30 s during the run. The data was analyzed using MassLynx V4.2 software from Waters.

Separation was carried out on a Luna RP-C18 column (150 mm × 4.6 mm i.d. × 4 µm, Phenomenex, Torrance, CA, USA) with a pre-column filter (Phenomenex, Torrance, CA, USA). Solvents used in the mobile phase were water with 0.1% formic acid (A) and acetonitrile with 0.1% formic acid (B). Then, 1 μL of sample was injected with a flow rate of 0.4 mL/min at 40 °C. The chromatographic gradient started at 75%A and 25% B, changing to 35% A and 65% B at 15 min, then to 15% A and 95% B, at 35 min, holding it for 2 min. Then, the column was equilibrated for 5 min to initial conditions.

### 2.4. Folin–Ciocalteu Determination

The determination was performed through a modified Singleton and Rossi method, employing the Folin–Ciocalteu (FC) reagent, which is composed of a mixture of phosphotungstic and phosphomolybdic acids. As previously reported [22], the assay comprises mixing 10 mL of Na_2_CO_3_ (7.5%) and 0.5 mL of FC reagent with 0.5 mL of the respective *C. longa* extract previously prepared in acidified MeOH (0.1% HCl). Subsequently, the volume was completed to 25 mL with water. A blank was prepared following the same procedure using 0.5 mL of MeOH (0.1% HCl) in place of the extract. Both extract mixtures and the blank were kept in the dark for 1 h, and afterwards absorbance was measured at 750 nm. The absorbance measurements obtained were extrapolated in a gallic acid calibration curve to obtain Folin–Ciocalteu (FC) reducing capacity results, further expressed as mg gallic acid equivalents (GAE)/g of the extract. Each determination was performed in triplicate.

### 2.5. DPPH Antioxidant Activity

DPPH evaluation was performed as previously reported [23]. Initially, a solution of the reagent, 2,2-diphenyl-1-picrylhidrazyl (DPPH) (0.25 mM), was elaborated by employing methanol as the solvent. Then, 0.5 mL of the prepared DPPH solution were mixed with 1 mL of *C. longa* extract at different concentrations. These solutions were incubated at 25 °C in the dark for 30 min at room temperature. The DPPH absorbance was measured at 517 nm. In addition, blanks were elaborated for each concentration. Trolox was used as the control. The inhibition percentage was determined as shown in the following equation:

(1) $$\mathrm{Inhibition}\;\mathrm{percentage}\;\left(\%\right)=\frac{\left({\mathrm{Abs}}_{\mathrm{blank}}-{\mathrm{Abs}}_{\mathrm{sample}}\right)}{{\mathrm{Abs}}_{\mathrm{blank}}}\times 100$$

Abs_blank_ correspond to absorbance of the blank and Abs_sample_ correspond to sample absorbance. The inhibition percentage was plotted against the respective sample concentration to determine the IC_50_, which corresponds to the quantity of the sample required to reach the 50% radical-scavenging activity. Each sample was analyzed in three independent assays.

### 2.6. Nitric Oxide Scavenging Activity

Nitric oxide (NO) was produced from sodium nitroprusside (SNP) and rapidly transformed into nitrite, which is a stable product. The nitrite concentration was determined by the Griess reaction [24]. SNP (5 mM) was mixed with different concentrations of polyphenol extracts (8–100 µg/mL) and a control of SNP without polyphenols was also prepared. Trolox was used as the control. All the mixtures were prepared in 96-well plates and incubated for 60 min in direct light to enhance NO production. Later, the Griess reagent (1% sulfanilamide and 0.1% naphthylethylenediamine dihydrochloride in 2% H_3_PO_4_) was added, incubated for 6 min and the absorbance was read at 540 nm. Sample blanks were prepared for each polyphenol concentration. The percentage of the NO-scavenging activity of the extract was calculated with the following equation:

(2) $$\%\;\mathrm{NO}\;\mathrm{scaveging}\;\mathrm{activity}=\frac{100\times \left[\mathrm{Nitrites}\right]\mathrm{SNP}-\left[\mathrm{Nitrites}\right]\mathrm{sample}}{\left[\mathrm{Nitrites}\right]\mathrm{SNP}}$$

Finally, NO-scavenging activity was expressed as the amount of extract needed to reduce 50% of the NO that was generated by SNP (IC_50_). Samples were analyzed in triplicate.

### 2.7. Statistical Analysis

In order to evaluate if the total curcuminoid contents (TC) and individual CUR, DMC and BDM quantification determined by UPLC-DAD play a role in the antioxidant activity, a Pearson correlation analysis was performed between total and individual curcuminoid contents and FC, DPPH and NO results. One-way analysis of variance (ANOVA) with a Tukey post hoc as statistical test was applied to TC, CUR, DMC, BDM, FC, DPPH and NO findings to evaluate significant differences (*p* < 0.05) between samples analyzed in the present study. In addition, Principal Component Analysis (PCA) was performed to summarize the data obtained from *C. longa* curcuminoid extracts (*n* = 12) considering all seven variables previously mentioned. R (version 1.2.1335) statistical program was used to perform the statistical analyses.

## 3. Results and Discussion

### 3.1. Extraction from C. longa Rhizomes

Extraction results of the three main curcuminoids from *C. longa* rhizome NR-1 were determined through the Pressurized Liquid Extraction (PLE) method applying a factorial design as previously described in the Materials and Methods Section 2.2. The efficiency of the extraction was evaluated through quantification of curcumin (CUR), demethoxycurcumin (DMC) and bisdemethoxycurcumin (BDM) by UPLC-DAD to yield the total curcuminoids contents (TC) in the sample. In the current study, a factorial 2^3^ design (FD) was employed to evaluate the effect of the three independent variables, namely solvent, temperature and extraction static time. The extractions were conducted under experimental conditions, as represented in Table 1. FD variables and levels were selected by considering previous results obtained for polyphenols extraction from food products [19]. For instance, PLE variables included 60 and 80 °C temperature; methanol and acetone as solvents and extraction static times of 6 and 10 min, corresponding within each factor to low (−1) and high (+1) levels respectively. The experiments were carried-out after randomization and every response was the average of two replicates.

Statistical analysis of the results did not show a significant effect (*p* < 0.05) for any variable, 2-way or 3-way interactions in the Half-normal plot of the effects and the Pareto chart. However, by excluding the 2-way interaction solvent-time showing the least effect, the FD findings indicated a significant difference (*p* < 0.05) for the temperature, corresponding to the highest standardized effect, with 80 °C yielding better results. In addition, 2-way interactions temperature-time and solvent-temperature also showed significant standardized effects (*p* < 0.05) with acetone and 10 min static time delivering better results. Finally, the cube plot indicated the highest fitted mean associated with the combination of these three levels, thus in agreement with the selection of the best conditions for curcuminoid extractions from *C. longa* rhizomes (*n* = 12), namely 80 °C of temperature, acetone as solvent and 10 min static time.

### 3.2. Polyphenolic Profile by UPLC-ESI-MS Analysis

**Figure 1:**
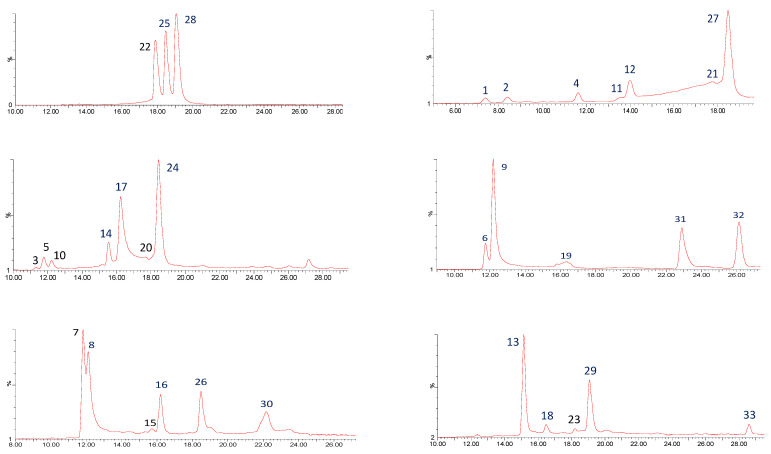
UHPLC QTOF-ESI MS extracted ion chromatograms of curcuminoids from *C. longa* rhizomes, in a Phenomenex Luna RP18 C-18 column (150 mm × 4.6 mm × 4 µm) using a Xevo G2-XS QTOF Mass spectrometer (Waters™, Wilmslow, UK) in a mass range from 100 to 1000 amu.

Through the UPLC-QTOF-ESI MS analysis described in the Materials and Methods section, 33 polyphenolic compounds were identified in Costa Rican *C. longa* rhizomes. Figure 1 shows the chromatograms for these compounds and Table 2 summarizes the analysis results for the 33 curcuminoids in the samples (n = 12).

The identified curcuminoids have a common main structure consisting of two phenolic rings connected by a central chain holding different functional groups as indicated in the following subsections.

#### 3.2.1. Curcuminoids with Keto Groups in C3 and C5

**Figure 2:**
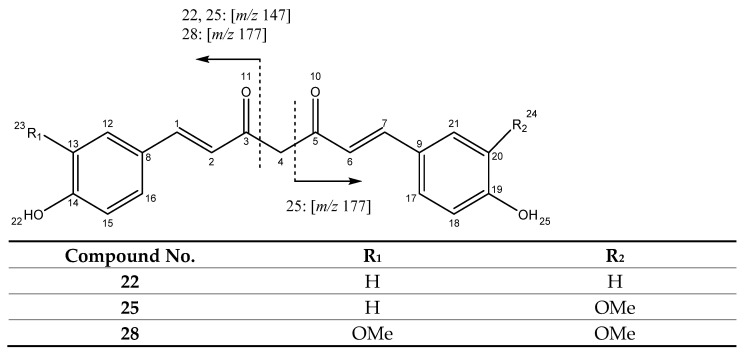
Structure and main fragments for compounds **22**, **25** and **28**.

The compounds in this group hold two keto moieties in C3 and C5, varying in the presence of double bonds in the central chain. The three best known curcuminoids [25] are included in this group. In fact, peaks 22 (Rt = 17.90 min), 25 (Rt = 18.46 min) and 28 (Rt = 19.03 min), were tentatively assigned to bisdemethoxycurcumin (BDM), demethoxycurcumin (DMC) and curcumin (CUR) based on the pseudomolecular ions [M + H]^+^ at *m/z* 309.1137 (C_19_H_17_O_4_), 339.1262 (C_20_H_19_O_5_) and 369.1358 (C_21_H_21_O_6_), respectively. The characteristic fragmentation pathway includes fragments at *m/z* 177, for curcumin and demethoxycurcumin, as well as at *m/z* 147 for bisdemethoxycurcumin and demethoxycurcumin, as shown in Figure 2.

A characteristic product ion is constituted by the neutral loss of carbon monoxide (28 uma) from the fragment at *m/z* 147, which gives rise to the fragment observed at *m/z* 119. On the other hand, the fragment at *m/z* 177 suffers a neutral loss of CH_3_OH (32 uma) to produce the fragment at *m/z* 145, which is observed for hydroxyl and methoxy groups in ortho position in the benzene ring, as found in curcumin and demethoxycurcumin [26,27].

**Figure 3:**
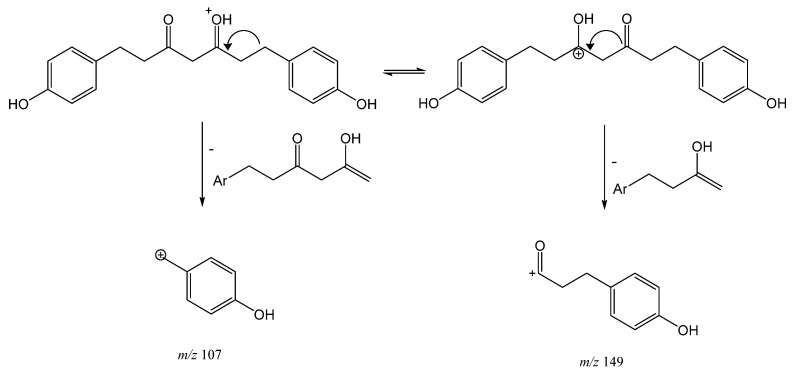
Fragmentation pathway of compound **4**.

Peak 4 (Rt = 11.60 min) with [M + H]^+^ at *m/z* 313.1422 (C_19_H_21_O_4_), which was tentatively assigned to tetrahydrobisdemethoxycurcumin, also holds a diketone moiety, but lacks double bonds in the central chain. This compound shows two common fragmentation pathways from the initial structure with a positive charge in C3, yielding product ion at *m/z* 149 [M + H−164]^+^ and another fragment at *m/z* 107 [M + H−206]^+^ (Figure 3), due to keto enol tautomerization, which is characteristic of positive ionization [26].

**Figure 4:**
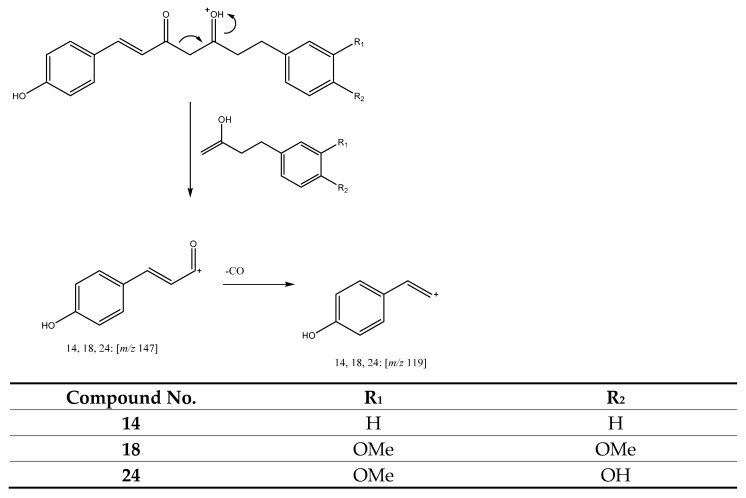
Fragmentation pathway of compounds **14**, **18** and **24**.

Peaks 14 (Rt = 15.52 min) and 18 (Rt = 16.46 min) were tentatively assigned to 1-(4-hydroxyphenyl)-7-phenylhept-1-ene-3,5-dione at [M + H]^+^ at *m/z* 295.1313 (C_19_H_19_O_3_) and 7-(3,4-dimethoxyphenyl)-1-(4-hydroxyphenyl)hept-1-ene-3,5-dione at [M + H]^+^ at *m/z* 355.1512 (C_21_H_23_O_5_), respectively. Meanwhile, peak 24 (Rt = 18.43 min) at *m/z* 341.1379 (C_20_H_21_O_5_) was tentatively assigned to dihydrodemethoxycurcumin. All of these peaks hold a positive charge on C5, present a double bond between C1–C2 and show a product ion at *m/z* 147, which then undergoes a loss of carbon monoxide (28 uma) to produce the fragment observed at *m/z* 119, as shown in Figure 4 [27,28].

**Figure 5:**
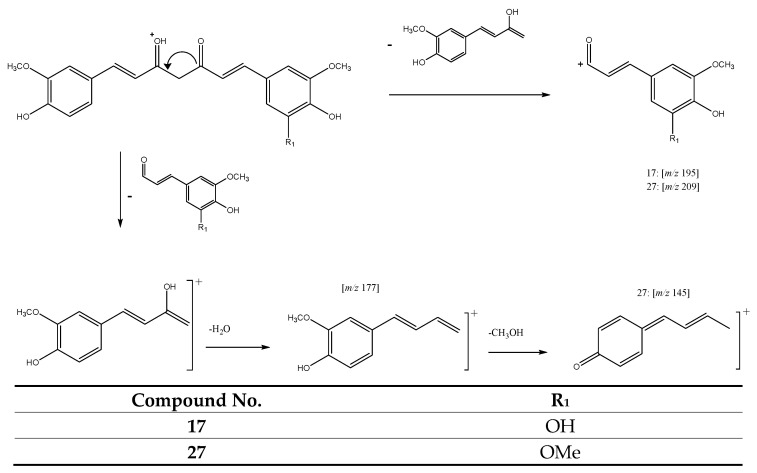
Fragmentation pathway of compounds **17** and **27**.

Finally, in this group of compounds, peaks 17 (Rt = 16.21 min) and 27 (Rt = 18.61 min) with [M + H]^+^ at *m/z* 385.1276 (C_21_H_21_O_7_) and *m/z* 399.1408 (C_22_H_23_O_7_) were tentatively assigned to curcumalongin C and 1-(4-hydroxy-3,5-dimethoxyphenyl)-7-(4-hydroxy-3-methoxyphenyl)-1,6-heptadiene-3,5-dione, respectively. Both peaks hold a positive charge in C3 and double bonds between C1-C2 and C6-C7. The fragmentation pathway for both molecules (Figure 5) yields a product ion at *m/z* 195 for peak 17 and *m/z* 209 for peak 27. On the other hand, the loss of these fragments followed by the loss of H_2_O yields for both compounds, a product ion at *m/z* 177, which in turn produces a fragment at *m/z* 145 corresponding to additional loss of MeOH [28,29].

#### 3.2.2. Curcuminoids with a Single Keto Moiety in C3

**Figure 6:**
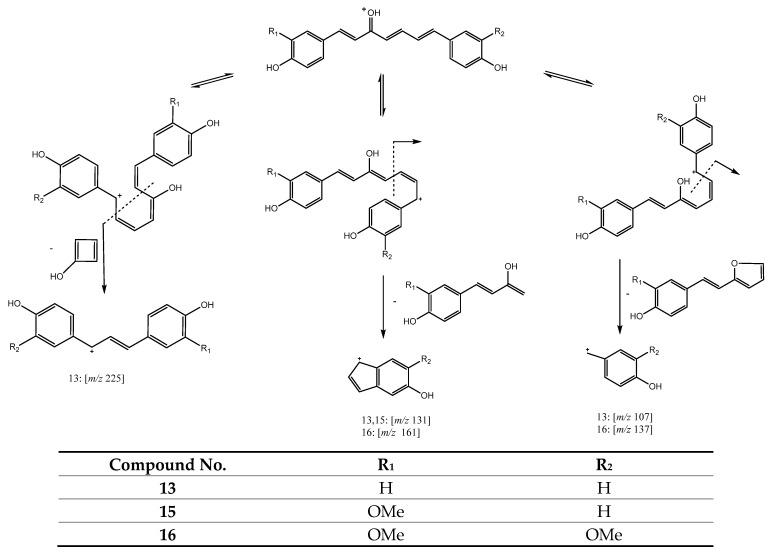
Fragmentation pathway for compounds **13**, **15**, **16**.

A second group of curcuminoids includes compounds with only one keto group in the central chain on C3. For instance, peak 13 (Rt = 15.13 min) with [M + H]^+^ at *m/z* 293.1167 (C_19_H_17_O_3_) and peak 15 (Rt = 15.67 min) with [M + H]^+^ at *m/z* 323.1253 (C_20_H_19_O_4_), are included in this group of compounds and were tentatively assigned to 1,7-bis(4-hydroxyphenyl)-1,4,6-heptatrien-3-one and 1-(4-hydroxy-3-methoxyphenyl)-7-(4-hydroxyphenyl)hepta-1,4,6-trien-3-one, respectively. In addition, peak 16 (Rt = 16.18 min) with [M + H]^+^ at *m/z* 353.1374 (C_21_H_21_O_5_), also belongs to this group and was assigned to 1,7-bis(4-hydroxy-3-methoxyphenyl)-1,4,6-heptatrien-3-one. These three compounds show a common fragmentation pathway involving rearrangement of the precursor ions with a hydrogen atom transfer and neutral loss of an aryl moiety to yield fragments at *m/z* 131 and *m/z* 161, as shown in Figure 6. A similar rearrangement occurs for peaks 13 and 16 with a neutral loss of a different aryl moiety including a heterocyclic five-member ring, which yields product ions at *m/z* 107 and *m/z* 137, respectively. Finally, peak 13 undergoes a specific rearrangement with the neutral loss of a four-member ring moiety to yield a fragment at *m/z* at 225, as shown in Figure 6 [26].

**Figure 7:**
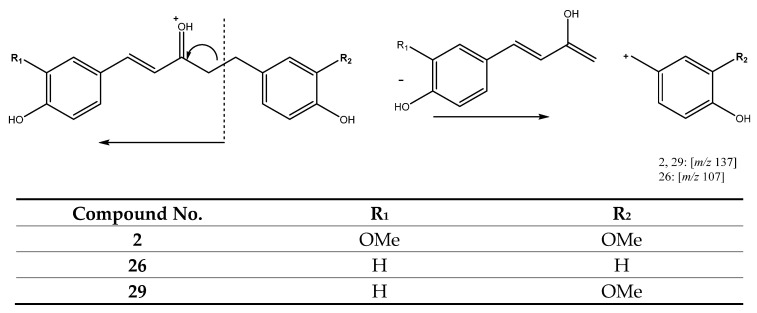
Fragmentation pathway for peaks **2**, **26** and **29**.

Further, peak 2 (Rt = 8.41 min) with [M + H]^+^ at *m/z* 329.1383 (C_19_H_21_O_5_), tentatively assigned to 1,5-bis(4-hydroxy-3-methoxyphenyl)pent-1-en-3-one and peak 26 (Rt = 18.51 min) with [M + H]^+^ at *m/z* 269.1168 (C_17_H_17_O_3_), tentatively identified as artamenone, belong to this group. In addition, peak 29 (Rt = 19.07 min) with [M + H]^+^ at *m/z* 299.1281 (C_18_H_19_O_4_), was also tentatively assigned to 5-(4-hydroxy-3-methoxyphenyl)-1-(4-hydroxyphenyl)pent-1-en-3-one. These compounds present similar skeletons, but with only five carbons in the aliphatic chain and a double bond in C1-C2. Their common fragmentation pathway includes rearrangement of the precursor ion with a hydrogen atom transfer and neutral loss of an Ar-C_4_H_5_O moiety, as shown in Figure 7 [26].

**Figure 8:**
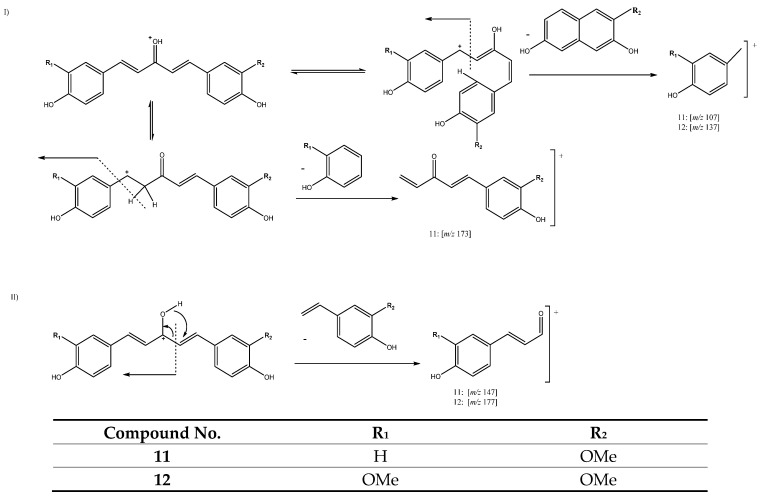
FFragmentation pathways I and II for compounds **11** and **12**.

Peaks 11 (Rt = 13.52 min) and 12 (Rt = 13.97 min) also pertain to this group of curcuminoids, with [M + H]^+^ at *m/z* 297.1105 (C_18_H_17_O_4_) and *m/z* 327.1216 (C_19_H_19_O_5_), tentatively identified as 1-(4-hydroxy-3-methoxyphenyl)-5-(4-hydroxyphenyl)penta-1,4-dien-3-one and 1,5-bis(4-hydroxy-3-methoxyphenyl)-1,4-pentadien-3-one, respectively. In fact, these two compounds also have five carbons in their aliphatic chain and a single keto group in C3 but with two double bonds between C1-C2 and C4-C5. They follow two different pathways of fragmentation, as shown in Figure 8. Pathway I is attributed to a reorientation of the molecule that produces the fragments at *m/z* 107 and *m/z* 137 for peaks 11 and 12 respectively, while peak 11 suffers the loss of a neutral aryl moiety to yield the fragment at *m/z* 173 (Figure 8). The other fragmentation pathway (II) is due to a hydrogen transfer, which in turn produces the ion products at *m/z* 147 and *m/z* 177, corresponding to peaks 11 and 12, respectively [27,29].

#### 3.2.3. Curcuminoids with a Keto Moiety in C3 and Hydroxyl in C5

**Figure 9:**
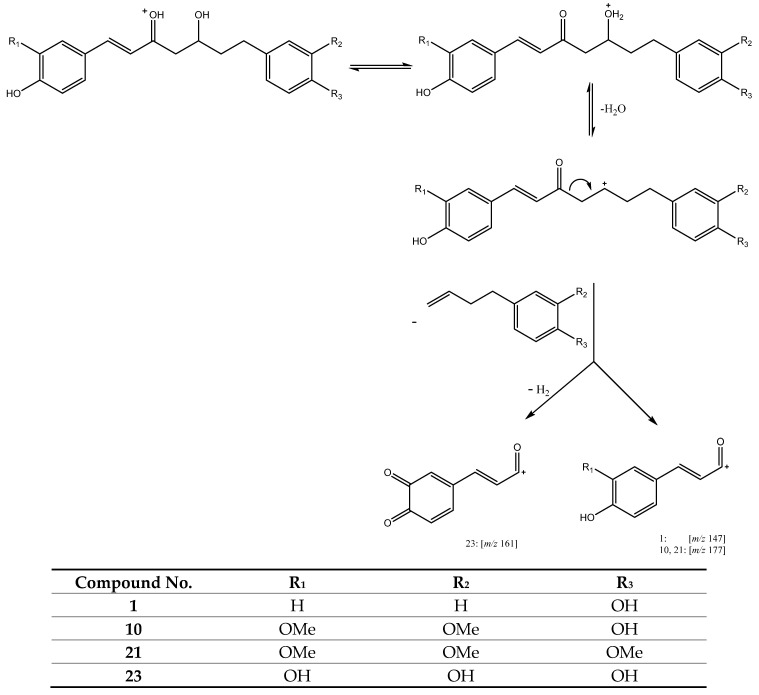
Fragmentation pathway of compounds **1**, **10** and **23**.

A third group of compounds includes compounds with a ketone in C3 and hydroxyl group in C5, and contain peak 1 (Rt = 7.39 min) with [M + H]^+^ at *m/z* 313.1422 (C_19_H_21_O_4_) tentatively assigned to 5-hydroxy-1,7-bis(4-hydroxyphenyl)hept-1-en-3-one, peak 10 (Rt = 12.39 min) with [M + H]^+^ at *m/z* 373.1652 (C_21_H_25_O_6_), tentatively identified as 5-hydroxy-1,7-bis(4-hydroxy-3-methoxyphenyl)hept-1-en-3-one, and peak 21 (Rt = 17.75 min) with [M + H]^+^ at *m/z* 387.1802 (C_22_H_27_O_6_), which was tentatively identified as 7-(3,4-dimethoxyphenyl)-5-hydroxy-1-(4-hydroxy-3-methoxyphenyl)hept-1-en-3-one. In addition, peak 23 (Rt = 18.18 min) with [M + H]^+^ at *m/z* 345.1336 (C_19_H_21_O_6_) was also tentatively assigned to 1,7-bis(3,4-dihydroxyphenyl)-5-hydroxyhept-1-en-3-one. These three compounds present a common pathway due the loss of the aryl group, as shown in Figure 9, to yield main fragments at *m/z* 147.04 for peak 1 and *m/z* 177.05 for peaks 10 and 21 [27]. While peak 23 suffers a subsequent dehydrogenation yielding a fragment at *m/z* 161 [26].

#### 3.2.4. Curcuminoids with Two Hydroxyl Groups in C3 and C5

**Figure 10:**
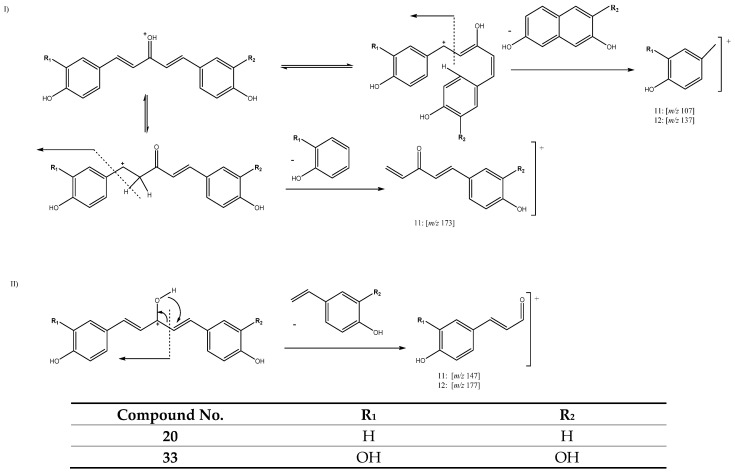
Fragmentation pathways I and II for compounds **20**, **33**.

The fourth group of compounds includes peak 20 (Rt = 17.25 min) with [M + H]^+^ at *m/z* 317.1733 (C_19_H_25_O_4_), tentatively assigned to octahydrobisdemethoxycurcumin and peak 33 (Rt = 28.14 min) with [M + H]^+^ at *m/z* 349.1640 (C_19_H_25_O_6_), tentatively assigned to 4,4′-(3,5-dihydroxyheptane-1,7-diyl)bis(benzene-1,2-diol), both with hydroxyl groups in C3 and C5 of the central chain. These two compounds yield fragments at *m/z* 107, 147 and 163 due to the fragmentation pathways including loss of neutral aromatic and olefin moieties as well as loss of water, as shown in Figure 10 [27].

#### 3.2.5. Curcuminoids with an Ester Group in C3 and Keto Moiety in C5

**Figure 11:**
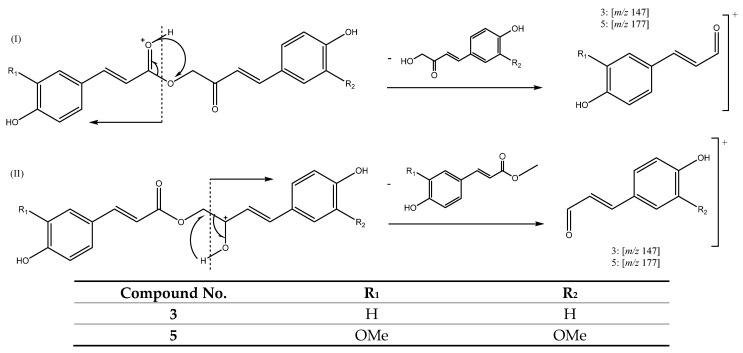
Fragmentation pathways I and II for compounds **3** and **5**.

A different group of curcuminoids that holds an ester in C3 and a keto group in C5, includes for instance peak **3** (Rt = 11.47 min), which was tentatively assigned to 4-(4-hydroxyphenyl)-2-oxobut-3-en-1-yl 3-(4-hydroxyphenyl)acrylate with [M + H]^+^ at *m/z* 325.1075 (C_19_H_17_O_5_). This group of compounds also includes peak 5 (Rt = 11.71 min), which was assigned to previously reported Calebin A [30] with *m/z* 385.1276 (C_21_H_21_O_7_). These compounds follow two different fragmentation patterns, as shown in Figure 11. Pathway I comprise a hydrogen rearrangement in the ester group followed by the neutral loss of an aryl group yielding ion products at *m/z* 147 for peak 3 and at *m/z* 177 for peak 5. The other fragmentation pathway (II) follows a similar pattern but with a hydrogen rearrangement in the keto group followed by the neutral loss of an aryl group also yielding fragments at *m/z* 147 for peak 3 and at *m/z* 177 for peak 5 [26].

#### 3.2.6. Curcuminoids with a Ring in the Central Chain

**Figure 12:**
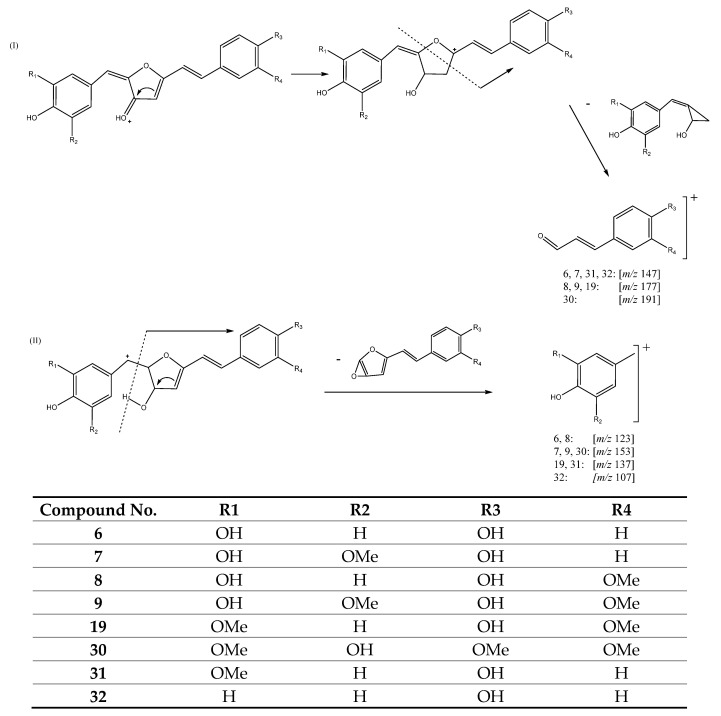
Fragmentation pathways I and II for compounds **6**, **7**, **8**, **9**, **19**, **30**, **31** and **32**.

The last group of curcuminoids presents a five-member ring in its structure, formed by a bond between C2 and the oxygen in C5. For instance, peaks 7 (Rt = 11.83 min) and 9 (Rt = 12.19 min), were tentatively assigned to curcumalongin A and 2-(3,4-dihydroxybenzylidene)-5-(4-hydroxy-3-methoxystyryl)furan-3(2H)-one with [M + H]^+^ at *m/z* 353.1024 (C_20_H_17_O_6_) while peak 8 (Rt = 12.11 min) with [M + H]^+^ at *m/z* 383.1140 (C_21_H_19_O_7_), was tentatively identified as curcumalongin B. Figure 12 shows characteristic fragmentation pathways for these compounds. Pathway I entails rearrangement of the precursor ions followed by cleavage of the furan ring to yield product ions at *m/z* 147 for peak 7 and at *m/z* 177 for peaks 8 and 9. In turn, pathway II involves a rearrangement of precursor ions via a γ-hydrogen shift and loss of an aryl epoxide moiety to deliver product ions at *m/z* 123 for peak 8 and at *m/z* 153 for peaks 7 and 9 [26].

Three other curcuminoids that belong to this group include peak 6 (Rt = 11.74 min) with [M + H]^+^ at *m/z* 323.0914 (C_19_H_15_O_5_), peak 31 (Rt = 22.88 min) with [M + H]^+^ at *m/z* 337.1054 (C_20_H_17_O_5_) and peak 32 (Rt = 26.16 min) with [M + H]^+^ at *m/z* 307.0948 (C_19_H_15_O_4_). These peaks were tentatively identified as 2-(3,4-dihydroxybenzylidene)-5-(-4-hydroxystyryl)furan-3(2H)-one, 2-(4-hydroxy-3-methoxybenzylidene)-5-(-4-hydroxystyryl) furan-3(2H)-one and 2-(4-hydroxybenzylidene)-5-(-4-hydroxystyryl)furan-3(2H)-one, respectively. As shown in Figure 12, Pathway I for these peaks yield the same product ion at *m/z* 147 while pathway II corresponding to the γ-hydrogen shift rearrangement and loss of the aryl epoxide moiety yields three different product ions at *m/z* 123, 137 and 107 for peaks 6, 31 and 32, respectively [26].

Finally, this last group of compounds also includes peak 19 (Rt = 16.56 min) with [M + H]^+^ at *m/z* 367.1176 (C_21_H_19_O_6_), which was tentatively identified as 2-(4-hydroxy-3-methoxybenzylidene)-5-(-4-hydroxy-3-methoxystyryl)furan-3(2H)-one and peak 30 (Rt = 22.15 min) with [M + H]^+^ at *m/z* 397.1262 (C_22_H_17_O_6_) that was tentatively assigned to 2-(3,4-dihydroxy-5-methoxybenzylidene)-5-(-3,4-dimethoxystyryl)furan-3(2H)-one. Rearrangement of these two precursor ions followed by cleavage of the furan ring along pathway I yield fragments at *m/z* 177 for peak 19 and at *m/z* 191 for peak 30. Meanwhile, the rearrangement via a γ-hydrogen shift and the subsequent loss of an aryl epoxide moiety produces a fragment at *m/z* 137 for peak 19 and at *m/z* 153 for peak 30 [26].

Compared with the literature, the findings in these 12 turmeric samples from Costa Rica are in agreement with previous reports on structures diversity accounting for the different compounds found in this study, including structures with an open aliphatic chain or those possessing a cyclic moiety [26,27,28,29].

### 3.3. Total Curcuminoid Contents in C. longa Extracts

UPLC-DAD analysis allowed the quantification of CUR, DMC and BDM as well as the determination of total curcuminoid (TC) contents, with results summarized in Table 3.

**Figure 13:**
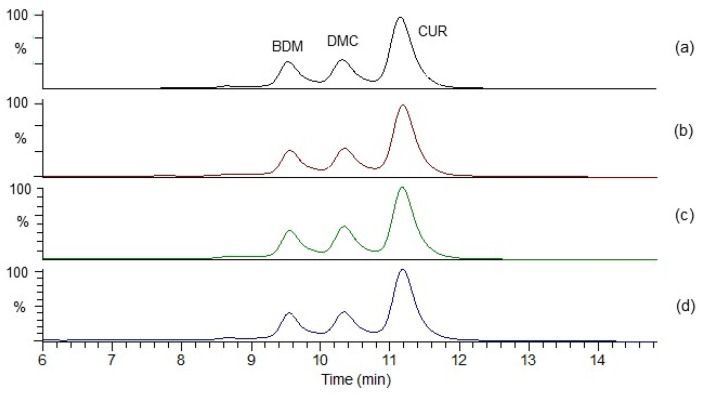
UPLC-DAD chromatograms for samples: (**a**) NE-1, (**b**) NR-1, (**c**) NW-1 and (**d**) WR-1, showing the three quantified curcuminoids CUR, DMC and BDM.

Figure 13 illustrates UPLC-DAD chromatograms for samples from the four different regions, showing the three quantifiable curcuminoids, CUR, DMC and BDM. Results for TC contents show values ranging from 49.9 mg/g dry material to 125.0 mg/g dry material, representing 4.9–12.5% content, thus higher than the 3% established by USP [21]. Samples from the Northeastern region (NE) show again the highest values with an average TC content of 107.3 mg curcuminoids/g dry material while the lowest values corresponded to turmeric samples from the Northern region (NR), which present an average of 73.3 mg curcuminoids/g dry material, thus 32% lower. At individual level, NR-4 exhibits the lowest value (49.9 mg/g dry material) among all 12 samples while NE-1 displays the highest value (125.0 mg/g dry sample), followed by NW-3 (118.7 mg/g dry material).

Reports from the literature show variability on total curcuminoid contents (TC). For instance, studies on turmeric rhizomes from China [31] and Thailand [32] report values ranging between 5.9 and 28.3 mg/g dry material, thus lower than TC results for samples in the present study. In turn, results for rhizomes from India [33] report TC values between 1.4 and 51.2 mg/g dry material, thus only sample NR-4 shows a value within this range while the other eleven Costa Rican samples exhibit higher content. In addition, at extract level, findings for the 12 samples range between 379.3 and 833.3 mg TC/g extract, which are within the range than those reported by other studies amounting to 435–751.1 mg TC/g extract for samples from India and Malaysia [34,35]. Noteworthy, samples NE-1 and NW-3 constitute especially enriched extracts with TC content of 814.1 and 833.3 mg/g extract, respectively.

**Figure 14:**
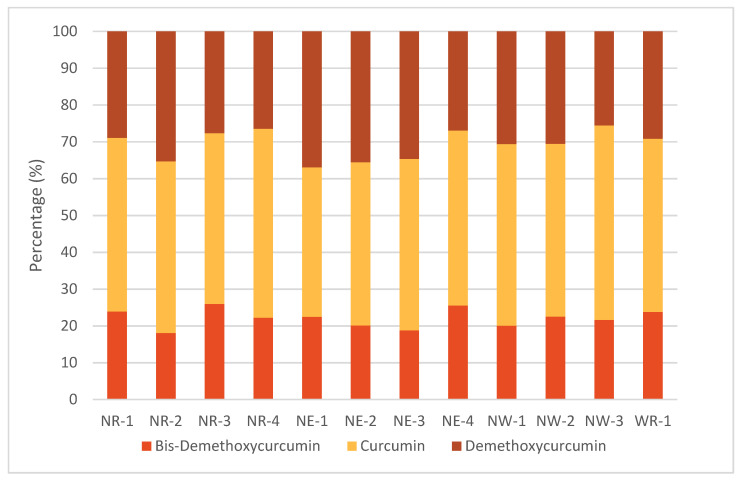
Percentage contents of CUR, DMC and BDM curcuminoids by UPLC-DAD for *C. longa* extracts.

Results for the 3 individual curcuminoids indicate that CUR exhibits the highest content in all 12 samples and BDM is the curcuminoid with the lowest content. For instance, CUR average value for all samples is 42.5 mg/g dry material compared to an average of 28.3 mg/g dry material for DMC and 19.7 mg/g material for BDM, thus accounting for CUR, showing 1.5- and 2.1-fold greater content, respectively. The distribution of CUR, DMC and BDM contents in samples exhibits variability, as shown in Figure 14, with NW-3 showing the highest percentage of CUR (52.8%), while NE-1 shows the highest percentage of DMC (37.0%) and NR-3 holds the highest percentage for BDM (26.0%). Secondary metabolites DMC and BDM show the highest percentage in NE-1, where they hold 59.4% of the TC content, while they show their lowest percentage in NW-3, where they represent 47.2% of the TC content.

Compared with the literature, these results are in agreement with CUR being the most abundant curcuminoid and with the curcuminoids distribution variability among samples [31,36]. For instance, the present results show the proportion of curcuminoids in the 12 turmeric rhizomes to be 40.6–52.9% for CUR, 25.5–35.3% for DMC and 18.1–26.0% for BDM while other studies in 14 rhizomes from China, India and Malaysia indicate that CUR accounts for 52.4–68.0%, DMC for 16.6–32.6% and BDM for 14.0–17.7% [31,34,35]. Finally, total content for secondary metabolites DMC and BDM for these rhizomes range between 32.0 and 46.8% while turmeric from Costa Rica present higher values of 47.2–59.4% for all 12 samples.

### 3.4. Folin–Ciocalteu Determination in C. longa Extracts

Recent studies [37,38] in polyphenols with different structures have demonstrated that Folin–Ciocalteu determination, widely used to assess total polyphenolic contents, is an adequate method to evaluate the polyphenolic reducing capacity, which is exerted through a single electron transfer mechanism [39,40]. Table 4 summarizes the results of applying the Folin–Ciocalteau method on PLE extracts from *C. longa* rhizomes (*n* = 12), as described in Section 2.4.

Results for total FC show values ranging from 214.8 mg of gallic acid equivalents (GAE)/g extract and 301.0 mg GAE/g extract. One-way ANOVA followed by Tukey post hoc test showed significant difference (*p* < 0.05) between samples from Northern and Northeastern regions. Samples from Northeastern region (NE) yield the highest results with an average FC value of 260.3 mg GAE/g extract in comparison to results for samples from the Northern region (NR), which exhibit an average of 229.3 mg GAE/g extract, thus 12% lower. At the individual level, NR-4 displays the lowest value (214.8 mg GAE/g extract) among all 12 samples followed by NW-1 (219.8 mg GAE/g extract) while NW-3 shows the highest value (301.0 mg GAE/g). This indicates high diversity between the samples from the Northwestern (NW) region while the samples from the Northeastern (NE) and Northern (NR) region display more homogeneous results. FC reducing capacity was also evaluated for Trolox standard, which presented a value of 13.91 mg GAE/mg of Trolox. Therefore, FC reducing capacity of extracts expressed as Trolox equivalents were found to be between 15.44 and 21.64 mg TE/g extract.

Previous studies from the literature have shown variability in the FC results for turmeric rhizomes. For instance, studies on samples from Malaysia [35] and Thailand [32] report values ranging from 221.7 to 317.6 mg GAE/g extract, thus similar to the results obtained in the present work, while other studies for samples from Korea [41] and India [33] report FC values between 2.6 and 10 mg GAE/g dry material, therefore lower than Costa Rican turmeric samples holding values ranging from 33.3 to 45.2 mg GAE/g dry material.

**Figure 15:**
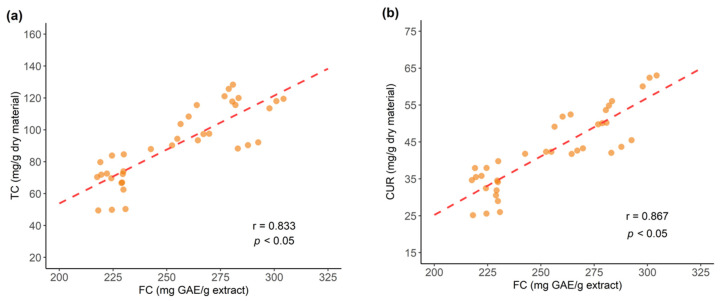
Correlation of Folin–Ciocalteu (FC) reducing capacity results and: (**a**) UPLC-DAD total curcuminoids (TC), (**b**) UPLC-DAD curcumin (CUR) contents.

The UPLC analysis results for Total Curcuminoids (TC) are in agreement with Folin–Ciocalteu (FC) determinations, for instance sample NR-4 with the poorest content (49.9 mg/g dry material) also presented the lowest FC value and samples NW-3 and NE-1 with the highest content of 118.7 and 125.0 mg/g dry material, respectively, also displayed high FC results aligning with TC findings. Further, a correlation analysis was performed between the Folin–Ciocalteu (FC) results and UPLC-DAD total curcuminoid content (TC) in all samples (*n* = 12), as shown in Figure 15a, as well as between FC and the UPLC-DAD determination for individual curcuminoids (CUR, DMC and BDM) contents.

Findings showed positive correlation between FC and TC (r = 0.833, *p* < 0.05) and regarding the individual curcuminoids, results indicated high positive correlation between FC and CUR (r = 0.867, *p* < 0.05), as shown in Figure 15b. This result is in agreement with previous studies for FC, reporting that replacing a hydrogen for a methoxy group, an electron donor, promotes electron transfer, therefore increasing the reducing capacity of a molecule [37,42,43], which is the case of CUR structure holding two methoxy groups compared to DMC with only one methoxy group and BDM that does not have any methoxy groups (Figure 2).

### 3.5. DPPH Antioxidant Activity

The capacity of scavenging free radicals can be conveniently assessed through the reaction with a stable free radical such as 2,2-diphenyl-1-picrylhidrazyl (DPPH) [44]. Kinetic studies for this assay have shown that the rate-determining step involves a fast electron transfer from phenoxide anions to DPPH, thus this reaction in protic organic solvents follows an electron transfer mechanism [45]. In order to perform the evaluation of DPPH assay in *C. longa* rhizomes (*n* = 12), the method was applied as described in the Materials and Methods Section 2.5, and results are presented in Table 5.

The findings for the DPPH antioxidant activity assessment indicate the same trend observed for the Folin–Ciocalteu reducing capacity determination (FC) and UPLC total curcuminoids (TC) contents, with samples NW-3 and NE-1 showing the lowest values, 16.07 and 15.21, respectively, therefore exhibiting higher antioxidant activity. One-way ANOVA followed by a Tukey post-hoc test showed significant difference (*p* < 0.05) for samples from the Northern (NR) and Northeastern (NE) regions. In fact, samples NR-1 to NR-4 presented the highest values with an average IC_50_ of 25.42 μg/mL, thus representing lower antioxidant activity. In contrast, samples NE-1 to NE-4 showed the lowest average IC_50_ of 18.22 μg/mL, corresponding to higher antioxidant activity. These observations are consistent with the results obtained for FC and TC. DPPH antioxidant activity of Trolox standard was also evaluated, exhibiting an IC_50_ of 5.62 μg/mL. Therefore, DPPH antioxidant activity for *Z. officinale* extracts expressed as Trolox equivalents delivered IC_50_ values between 0.19 and 0.37 μg TE/mL.

Compared with the literature, results reported for samples from India [33] and Thailand [32] range between 78.17 and 294.8 µg dry material/mL, in a similar range as samples in the present study, which range between 101.4 and 194.1 µg dry material/mL. Rhizomes from other curcuma species have shown variability for DPPH antioxidant activity evaluation, for instance *Curcuma amada* rhizomes extracts presented an IC_50_ of 22.01 µg/mL [46], similar to the present results while *Curcuma caesia* rhizomes exhibited an IC_50_ of 94 µg/mL [47], thus showing lower antioxidant activity than Costa Rican samples.

At an individual level, sample NW-3 presented the highest antioxidant activity (IC_50_ 15.21 μg/mL), followed by NE-1 (IC50 16.07 μg/mL) and NE-4 (IC50 18.51 μg/mL). These results suggest that not only the TC content is important for the antioxidant activity, but it might also be influenced by the percentage of CUR present. For instance, NW-3 has the highest percentage of CUR (52.8%) compared to NE-1 (40.6%) and NE-4 (47.5%). This would be consistent with results previously reported for the antioxidant activity of the main curcuminoids that indicated CUR as the major contributor to antioxidant potential [48].

**Figure 16:**
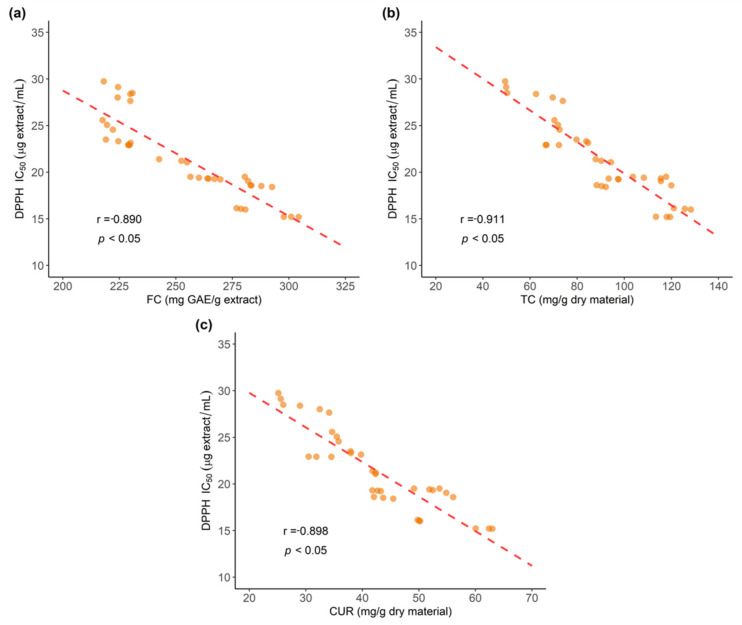
Correlation of antioxidant activity assessed by the DPPH method and: (**a**) Folin–Ciocalteu (FC) results; (**b**) Total curcuminoids (TC) content measured by UPLC-DAD; and (**c**) curcumin (CUR) content measured by UPLC-DAD.

Correlation analysis between DPPH antioxidant activity and Folin–Ciocalteu reducing capacity results (FC) as well as with UPLC-DAD total curcuminoids (TC) content were performed, as shown in Figure 16.

Results show significant negative correlation (*p* < 0.05) for DPPH values and Folin–Ciocalteau (FC) results (r = −0.890). In addition, DPPH results and TC content from UPLC-DAD quantification also show significant high negative correlation (r = −0.911, *p* < 0.05). These results agree with previous reports showing correlation for DPPH and FC results [49,50].

Further, correlation between DPPH values and individual curcuminoids determined by UPLC-DAD was also evaluated. A significant negative correlation was displayed for DPPH results and CUR contents (r = −0.898, *p* < 0.05), as shown in Figure 16. This result aligns with the fact that methoxy groups facilitate electron transfer, as mentioned earlier [37,42,43], thus the reducing capacity is increased by the presence of the two methoxy groups in CUR compared to one in DMC and none in BDM.

### 3.6. Nitric Oxide Radical Scavenging Activity

Nitric oxide (NO) has an important role as a bioregulatory molecule required for physiological processes such as immune response, blood pressure, vasodilatation, and neural signal transmission. However, the outcomes due to an excess of NO can result in an inflammatory context associated with different pathologies including, for instance, cancer, diabetes, and cardiovascular diseases. [51,52,53]. Hence, it is important to evaluate the potential to counteract NO formation through radical scavenging, in order to prevent the negative effects on the immune system and health caused by NO excess. The evaluation of the antioxidant activity of *C. longa* rhizomes (*n* = 12) through the NO assay was performed as described in the Materials and Methods Section 2.6, and results are presented in Table 6.

**Figure 17:**
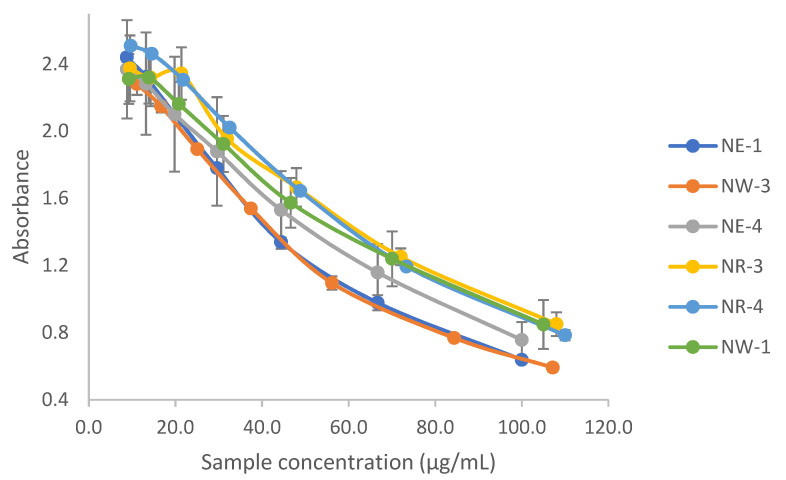
NO scavenging activity of selected samples (*n* = 6) of extracts from *C. longa* rhizomes.

NO scavenging activity results, expressed as IC_50_, range from 52.5 μg/mL to 81.9 μg/mL. As illustrated in Figure 17 as well, the lowest IC_50_, therefore the highest scavenging activity, corresponds to sample NW-3 (IC_50_ 52.5 μg/mL), followed by NE-1 (IC_50_ 54.3 μg/mL) and NE-4 (IC_50_ 65.5 μg/mL) in agreement with results for DPPH assay with the best antioxidant values for these three samples. On the other hand, the lowest scavenging activity corresponds to samples NR-3 (IC_50_ 81.9 μg/mL), followed by NW-1 (IC_50_ 79.2 μg/mL) and NR-4 (IC_50_ 78.5 μg/mL), which were also the three samples with lower antioxidant activity in the DPPH assay. NO scavenging activity for Trolox standard was also evaluated, showing an IC_50_ of 39.50 μg/mL. Results for extracts expressed as Trolox equivalents were between 0.51 and 0.75 μg TE /mL. 

Compared with previous results on the effect of curcuminoid extracts from *C. longa* rhizomes on NO scavenging activity, samples NE-1 and NW-3 are within the range of a reported IC_50_ value of 39 μg/mL for turmeric from Slovakia [54]. On the other hand, NO scavenging activity obtained for curcuminoids extracts from rhizomes of other *Curcuma* species indicated variability in results with IC_50_ ranging from 7.18 μg/mL for *Curcuma amada* [46] and 155 μg/mL for *Curcuma caesia* [47], therefore all 12 turmeric samples analyzed in the current study present scavenging activity values between these two curcuma species.

In turn, turmeric samples from the current study showed higher NO scavenging results than other roots and stems of plants with medicinal properties, for instance, extracts from *Rubia cordifolia* yielded IC_50_ values ranging from 94.53 and 153.7 μg/mL [55,56], while extracts from *Lantana camara* and from *Ventilago madraspatana*, traditionally used in inflammatory diseases, showed IC_50_ of 145.3 μg/mL and 168.3 μg/mL, respectively [56]. Further, IC_50_ values of present turmeric samples were shown to be within the range of rhizome extracts of *Cnidium officinale* (IC_50_: 57.25 μg/mL) and *Ligusticum chuanxiong* (IC_50_: 76.50 μg/mL) [57]. Finally, in comparison to fruit extracts, turmeric samples in this study exhibit higher scavenging activity in respect to *Averrhoa bilimbi* (IC_50_: 85.01 μg/mL) [58] and *Limonia acidissima* (IC_50_: 70–125 μg/mL) [59] fruit extracts, while showing lower NO scavenging activity compared to blackberry phenolic extracts, which yielded an IC_50_ of 24.5 μg/mL [60].

**Figure 18:**
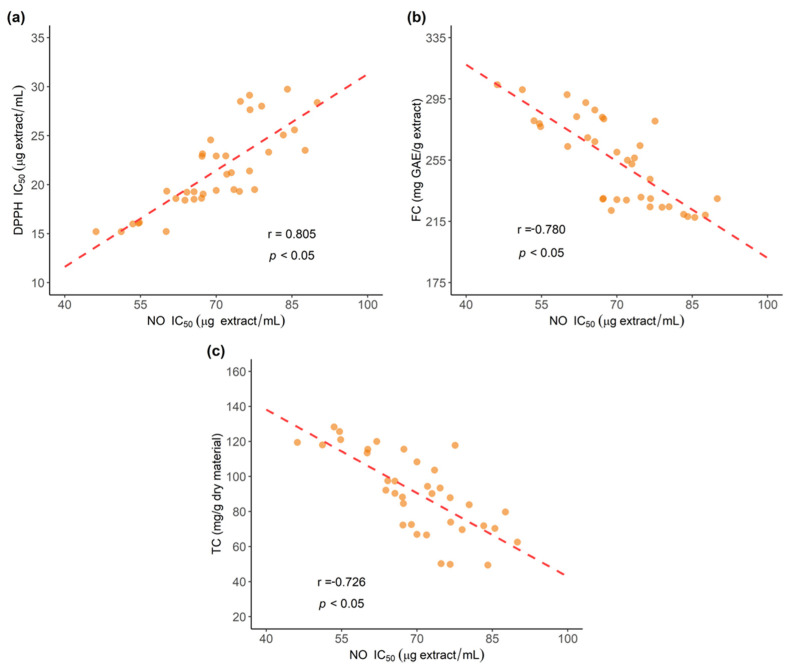
Correlation of antioxidant activity assessed through the Nitric Oxide (NO) radical scavenging and: (**a**) DPPH antioxidant activity (**b**) Folin–Ciocalteu (FC) reducing capacity results; (**c**) total curcuminoids (TC) content determined by UPLC-DAD.

A Pearson correlation study was performed to evaluate the relationship between NO scavenging activity results and the values obtained for DPPH antioxidant determination. As shown in Figure 18a, findings indicated positive correlation between NO results and DPPH antioxidant values (r = 0.805, *p* < 0.05), which aligns with previous results accounting for similarities among DPPH and NO assays, since their mechanisms involve direct methods and both use nitrogen radicals [60,61].

Further correlation studies between NO results with Folin–Ciocalteau (FC) reducing capacity results (Figure 18b) and UPLC total curcuminoids (TC) content (Figure 18c) were also performed, with findings indicating a negative correlation (*p* < 0.05) with FC and TC (r = −0.780 and r = −0.726, respectively). In respect to individual curcuminoids content, findings indicated similar contribution from CUR, DMC and BDM, all with low correlation values. These results are in agreement with previous results, evidencing that besides the methoxy and phenolic groups, the 1,3-diketone system might play an important role in nitric oxide (NO) scavenging, since all three curcuminoids showed similar NO scavenging activity [62,63].

In sum, correlation studies on polyphenols contents and antioxidant activities from literature show variability of results with some studies indicating low or no correlation [37,61], others reporting structure and assay dependence [64] while correlation is reported in several other studies [65,66,67]. The present findings for total curcuminoids (TC) content, FC reducing capacity, DPPH antioxidant activity and NO radical scavenging, considering mechanistic factors, are in agreement with the last group of studies reporting a correlation between antioxidant activity and polyphenols content, thus aligning with findings suggesting that these metabolites may play an important role in higher antioxidant capacity. Further, regarding correlation with the three main individual polyphenols, namely CUR, DMC and BDM (Figure 2), their UPLC-DAD content showing high correlation with FC and DPPH for CUR align with previous findings on the contribution of the methoxy groups present in CUR to the reducing capacity [37,42,43]. Instead, in the case of nitric oxide radical scavenging activity, the similar contribution from the three curcuminoids is in agreement with previous evidence pointing to the 1,3-diketone system, which is common in all three molecules, playing an important role in NO scavenging besides the methoxy and phenolic groups [62,63].

Despite these promising results for *C. longa* extracts, the antioxidant assays performed are non-physiological models, and although other polyphenolic extracts have shown strong correlation between these types of antioxidant assays and cellular antioxidant capacity [68], in vitro and in vivo model studies are needed to validate the extracts bioactive properties on cells. For instance, nano-encapsulated curcuminoids have shown important cellular antioxidant capacity [69], implying the possible use of these formulations aiming to increase the bioavailability of curcuminoids.

### 3.7. Principal Component Analysis for Polyphenolic Extracts of C. longa Rhizomes

In order to summarize the results, a Principal Component Analysis (PCA) was performed for *C. longa* rhizomes (*n* = 12) considering seven variables, namely TC, CUR, DMC, BDM, FC, DPPH and NO values. Two components (PC1 and PC2) were obtained (loadings > 0.38). The first component (PC1) corresponded to 84.10% of total variance and presented a negative correlation with TC, CUR, FC and positive correlation with DPPH. The second component (PC2) described 6.95% of the total variance and had a positive correlation with DMC quantification results.

**Figure 19:**
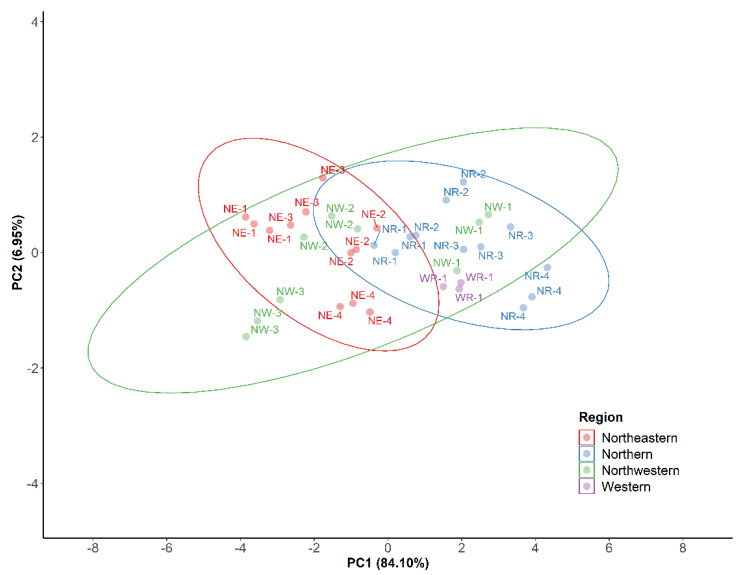
Graphic representation of the plane defined by the two first principal components (PC1 and PC2) deriving from the PCA analysis of *C. longa* rhizomes (*n* = 12) contents. Regions: Northeastern (NE), Northern (NR), Northwestern (NW), Western (WR).

As shown in the graphic representation of the plane defined by the two components (Figure 19), *C. longa* samples are distributed along PC1, corresponding to variability for the above-mentioned polyphenolic contents and DPPH antioxidant activity. For instance, PCA shows sample NR-4 holding the highest PC1 value, therefore accounting for the lowest TC, CUR and FC values as well as for the highest DPPH value, thus representing the lowest antioxidant activity.

In turn, some samples present much lower values in PC1, namely NW-3 and NE-1, which corresponds to higher FC, TC and CUR contents and lower DPPH values, thus accounting for greater antioxidant activity among all 12 samples. On the other hand, regarding PC2, sample NW-3 shows the lowest PC2, corresponding to its low DMC content amid extracts (*n* = 12), while samples NE-3, NR-2 and NE-1 show highest PC2 in agreement with their higher DMC results.

Finally, PCA findings indicate that although differences are observed in the composition between products from different regions, samples from the Northern (NR) and Northeastern (NE) regions deliver more homogeneous results while samples from the Northwestern (NW) region present more diversity. Noteworthy, samples NE-1 and NW-3 stand out significatively, showing the lowest PC1 value due to their rich total content of curcuminoids and high antioxidant activity.

## 4. Conclusions

*C. longa* rhizomes (*n* = 12) from Costa Rica show high diversity of polyphenols and important contents of curcuminoids as well as PCA, indicating especially higher contents and antioxidant activity for all samples from the Northeastern (NE) region, therefore suggesting the potential advantage of their homogeneity for obtaining standardized bioactive products from these rhizomes.

Curcuminoid extracts evaluated in this paper clearly exhibit a potential benefit concerning their capacity to protect against oxidative stress, due to their antioxidant activity values. Therefore, the promotion of these products as functional food and their consumption as dietary supplements could be beneficial for human health.

Nonetheless, further studies are required to assess their bioactive properties, for instance using in vitro cellular studies for antioxidant activity as well as exploring options to overcome their limited bioavailability for instance through the elaboration of nano-formulations.

## Figures and Tables

**Table 1:** UPLC-DAD quantification of total curcuminoids in turmeric sample NR-1 under various PLE conditions.

Experiment	Solvent	T (°C)	Static Time (min)	TC ^1,2,3^
1	Methanol	80	10	88.6 ^a,b^ ± 1.1
2	Acetone	80	10	90.8 ^a^ ± 3.2
3	Acetone	80	6	86.6 ^a,b^ ± 1.3
4	Methanol	80	6	81.0 ^b^ ± 0.8
5	Methanol	60	6	65.3 ^c^ ± 2.8
6	Acetone	60	6	57.8 ^c,d^ ± 3.7
7	Acetone	60	10	51.9 ^d^ ± 4.7
8	Methanol	60	10	57.7 ^c,d^ ± 1.0

^1^ Total Curcuminoids contents (TC) is expressed as mg/g dry material ^2^ Values are expressed as mean ± standard deviation (S.D.). ^3^ Different superscript letters indicate differences are significant at *p* < 0.05 using one-way analysis of variance (ANOVA) with a Tukey post hoc as statistical test.

**Table 2:** Profile of the phenolic compounds identified by UPLC-QTOF-ESI MS in *Curcuma longa* rhizomes from Costa Rica.

Peak	Tentative Identification	Rt (min)	Molecular Formula	[M + H]^+^ Observed	MS2 Fragments	Sample ^1^
1	5-hydroxy-1,7-bis(4-hydroxyphenyl)hept-1-en-3-one	7.39	C_19_H_21_O_4_	313.1422	147, 163, 133, 107	NR-1, NR-2, NE-3, NE-4, NW-1
2	1,5-bis(4-hydroxy-3-methoxyphenyl)pent-1-en-3-one	8.41	C_19_H_21_O_5_	329.1383	137	NE-1, NE-2, NE-3, NE-4, NW-2, NW-3, WR-1
3	4-(4-hydroxyphenyl)-2-oxobut-3-en-1-yl 3-(4-hydroxyphenyl)acrylate	11.47	C_19_H_17_O_5_	325.1075	147	NR-1, NR-4, NE-1, NE-2, NE-3, NE-4, NW-3, WR-1
4	Tetrahydrobisdemethoxycurcumin	11.60	C_19_H_21_O_4_	313.1422	149, 107	NR-3, NE-1, NE-3, NE-4, WR-1
5	Calebin-A isomer	11.71	C_21_H_21_O_7_	385.1276	177	NR-2, NR-3, NE-1, NE-3, NE-4, NW-1, NW-2
6	2-(3,4-dihydroxybenzylidene)-5-(-4-hydroxystyryl)furan-3(2H)-one	11.74	C_19_H_15_O_5_	323.0922	123, 147	NR-2, NR-3, NR-4, NE-1, NE-3, NE-4, NW-2, NW-3, WR-1
7	curcumalongin A	11.83	C_20_H_17_O_6_	353.1024	147, 153, 171, 269, 293, 321, 338	NR-2, NR-3, NR-4, NE-1, NE-2, NE-4, NW-1, NW-3
8	curcumalongin B	12.11	C_21_H_19_O_7_	383.1140	123, 145, 153, 177, 201, 294, 350, 368	NR-2, NR-4, NE-1, NE-2, NE-4, NW-1, NW-3
9	2-(3,4-dihydroxybenzylidene)-5-(4-hydroxy-3-methoxystyryl)furan-3(2H)-one	12.19	C_20_H_17_O_6_	353.1024	123, 150, 153, 177, 337, 338	NR-1, NR-2, NR-3, NR-4, NE-1, NE-2, NE-3, NE-4, NW-1, NW-2, NW-3, WR-1
10	5-hydroxy-1,7-bis(4-hydroxy-3-methoxyphenyl)hept-1-en-3-one	12.39	C_21_H_25_O_6_	373.1652	145, 163, 177, 137	NR-1, NR-2, NR-3, NR-4, NE-1, NE-2, NE-3, NE-4, NW-1, NW-2, NW-3, WR-1
11	1-(4-hydroxy-3-methoxyphenyl)-5-(4-hydroxyphenyl)penta-1,4-dien-3-one	13.52	C_18_H_17_O_4_	297.1105	107, 119, 137, 145, 147, 173, 177	NR-1, NR-3, NE-1, NE-2, NE-4, NW-3
12	1,5-bis(4-hydroxy-3-methoxyphenyl)-1,4-pentadien-3-one	13.97	C_19_H_19_O_5_	327.1216	137, 145, 177	NR-1, NE-1, NE-2, NE-3, NE-4, NW-1, NW-2, NW-3, WR-1
13	1,7-bis(4-hydroxyphenyl)-1,4,6-heptatrien-3-one	15.13	C_19_H_17_O_3_	293.1167	107, 131, 147, 173, 199, 225	NR-1, NR-2, NR-4, NE-1, NE-2, NE-3, NE-4, NW-1, NW-2, WR-1
14	1-(4-hydroxyphenyl)-7-phenylhept-1-ene-3,5-dione	15.52	C_19_H_19_O_3_	295.1313	105, 119, 147	NR-1, NR-2, NR-3, NR-4, NE-1, NE-2, NE-4, NW-1, NW-2, NW-3, WR-1
15	1-(4-hydroxy-3-methoxyphenyl)-7-(4-hydroxyphenyl)hepta-1,4,6-trien-3-one	15.67	C_20_H_19_O_4_	323.1253	107, 131, 137, 161, 177, 229	NR-2, NR-3, NE-1, NE-2, NE-3, NE-4, NW-1, NW-3
16	1,7-bis(4-hydroxy-3-methoxyphenyl)-1,4,6-heptatrien-3-one	16.18	C_21_H_21_O_5_	353.1370	137, 145, 161, 177, 225	NR-1, NR-4, NE-1, NE-2, NE-3, NE-4, NW-2, NW-3
17	Curcumalongin C	16.21	C_21_H_21_O_7_	385.1276	117, 133, 145, 161, 177, 193, 195, 219	NR-1, NR-2, NE-1, NE-2, NE-3, NE-4, NW-2, NW-3, WR-1
18	7-(3,4-dimethoxyphenyl)-1-(4-hydroxyphenyl)hept-1-ene-3,5-dione	16.46	C_21_H_23_O_5_	355.1512	119, 147	NR-2, NE-1, NE-2, NE-4, NW-1
19	2-(4-hydroxy-3-methoxybenzylidene)-5-(-4-hydroxy-3-methoxystyryl)furan-3(2H)-one	16.56	C_21_H_19_O_6_	367.1176	137, 177, 201, 323	NR-1, NR-2, NR-3, NR-4, NE-1, NE-2, NE-3, NE-4, NW-1, NW-2, NW-3, WR-1
20	Octahydrobisdemethoxycurcumin	17.25	C_19_H_25_O_4_	317.1733	107, 147, 161, 281	NR-2, NR-3, NE-1, NE-2, NE-4, NW-1
21	7-(3,4-dimethoxyphenyl)-5-hydroxy-1-(4-hydroxy-3-methoxyphenyl)hept-1-en-3-one	17.75	C_22_H_27_O_6_	387.1826	145, 177, 219	NR-1, NR-3, NE-1, NE-4, NW-3
22	Bisdemethoxycurcumin	17.90	C_19_H_17_O_4_	309.1137	147, 225	NR-1, NR-2, NR-3, NR-4, NE-1, NE-2, NE-3, NE-4, NW-1, NW-2, NW-3, WR-1
23	1,7-bis(3,4-dihydroxyphenyl)-5-hydroxyhept-1-en-3-one	18.18	C_19_H_21_O_6_	345.1336	161, 149, 123, 147	NR-1, NR-2, NR-4, NE-1, NE-2, NE-3, NE-4, NW-1, NW-2, NW-3, WR-1
24	Dihydrodemethoxycurcumin	18.43	C_20_H_21_O_5_	341.1379	119, 145, 147, 177	NR-2, NR-3, NE-1, NE-4, NW-2, NW-3
25	Demethoxycurcumin	18.46	C_20_H_19_O_5_	339.1262	117, 119, 131, 145, 147, 177, 195, 223	NR-1, NR-2, NR-3, NR-4, NE-1, NE-2, NE-3, NE-4, NW-1, NW-2, NW-3, WR-1
26	Artamenone	18.51	C_17_H_17_O_3_	269.1168	119, 107	NR-2, NR-3, NE-1, NE-4, NW-1, NW-3
27	1-(4-hydroxy-3,5-dimethoxyphenyl)-7-(4-hydroxy-3-methoxyphenyl)-1,6-heptadiene-3, 5-dione	18.61	C_22_H_23_O_7_	399.1408	145, 147, 161, 177, 209	NR-1, NR-2, NR-4, NE-1, NE-2, NE-3, NE-4, NW-1, NW-2, NW-3
28	Curcumin	19.03	C_21_H_21_O_6_	369.1358	117, 145, 161, 177, 219, 225	NR-1, NR-2, NR-3, NR-4, NE-1, NE-2, NE-3, NE-4, NW-1, NW-2, NW-3, WR-1
29	5-(4-hydroxy-3-methoxyphenyl)-1-(4-hydroxyphenyl)pent-1-en-3-one	19.07	C_18_H_19_O4	299.1281	137	NR-1, NR-3, NE-4, NW-2, NW-3
30	2-(3,4-dihydroxy-5-methoxybenzylidene)-5-(-3,4-dimethoxystyryl)furan-3(2H)-one	22.15	C_22_H_21_O_7_	397.1262	191, 153	NR-1, NR-2, NR-4, NE-1, NE-2, NE-3, NE-4, NW-3
31	2-(4-hydroxy-3-methoxybenzylidene)-5-(-4-hydroxystyryl)furan-3(2H)-one	22.88	C_20_H_17_O_5_	337.1054	137, 147	NR-1, NR-2, NR-3, NR-4, NE-1, NE-2, NE-3, NE-4, NW-1, NW-2, NW-3, WR-1
32	2-(4-hydroxybenzylidene)-5-(-4-hydroxystyryl)furan-3(2H)-one	26.16	C_19_H_15_O_4_	307.0948	107, 147	NR-1, NR-2, NR-4, NE-1, NE-2, NE-3, NE-4, NW-1, NW-2, NW-3, WR-1
33	4,4′-(3,5-dihydroxyheptane-1,7-diyl)bis(benzene-1,2-diol)	28.14	C_19_H_25_O_6_	349.164	149, 163, 177	NE-1, NE-2, NE-4, NW-2, NW-3

^1^ Regions: Northern (NR), Northeastern (NE), Northwestern (NW), Western (WR).

**Table 3:** Total curcuminoid (TC) content in *C. longa* rhizomes.

Product	CUR (mg/g) ^1,2,3^	DMC (mg/g)	BDM (mg/g) ^1,2,3^	TC (mg/g) ^1,2,3^
NR-1	42.1 ^a,b^ ± 0.3	25.9 ^a,b^ ± 1.0	21.5 ^a,b,c^ ± 3.0	90.8 ^a,b^ ± 3.3
NR-2	38.6 ^b,c^ ± 1.0	29.3 ^a,c^ ± 2.1	15.0 ^d,e^ ± 1.0	82.7 ^b,c^ ± 2.6
NR-3	31.8 ^d^ ± 2.6	19.0 ^d^ ± 1.3	17.9 ^c,d^ ± 1.8	68.7 ^d^ ± 5.8
NR-4	25.6 ^e^ ± 0.4	13.2 ^e^ ± 0.1	11.1 ^e^ ± 0.1	49.9 ^e^ ± 0.5
NE-1	50.0 ^f^ ± 0.2	46.6 ^f^ ± 1.7	28.4 ^f^ ± 2.0	125.0 ^f^ ± 3.7
NE-2	42.6 ^a,b^ ± 0.8	34.2 ^c^ ± 1.2	19.4 ^b,c,d^ ± 0.5	96.1 ^a^ ± 2.4
NE-3	54.8 ^f^ ± 1.2	40.8 ^g^ ± 0.6	22.1 ^a,b,c^ ± 0.4	117.8 ^g^ ± 2.2
NE-4	43.7 ^a^ ± 1.7	25.1 ^a,b^ ± 2.4	21.5 ^a,b^ ± 1.2	90.3 ^a^ ± 2.0
NW-1	35.3 ^c,d^ ± 0.6	21.9 ^b,d^ ± 0.1	14.3 ^d,e^ ± 0.4	71.6 ^c,d^ ± 1.1
NW-2	51.2 ^f^ ± 1.8	33.4 ^c^ ± 3.8	24.6 ^a,f^ ± 2.0	109.1 ^g^ ± 6.0
NW-3	62.7 ^g^ ± 0.4	30.5 ^a,c^ ± 0.3	25.6 ^a,f^ ± 0.3	118.7 ^g^ ± 1.0
WR-1	32.3 ^d^ ± 2.0	20.0 ^d^ ± 1.0	16.3 ^d^ ± 0.6	68.6 ^d^ ± 3.2

^1^ mg of curcuminoid/g dry material. ^2^ Values are expressed as mean ± standard deviation (S.D.). ^3^ Different superscript letters in the same column indicate that differences are significant at *p* < 0.05 using one-way analysis of variance (ANOVA) with a Tukey post hoc as the statistical test.

**Table 4:** Folin–Ciocalteu (FC) reducing capacity results for extracts of *C. longa* rhizomes.

Product	FC (mg GAE/g) ^1,2,3^	Product	FC (mg GAE/g) ^1,2,3^
NR-1	250.0 ^a^ ± 6.5	NE-3	281.9 ^c^ ± 1.5
NR-2	224.5 ^b^ ± 5.4	NE-4	287.8 ^c^ ± 4.8
NR-3	228.0 ^b^ ± 5.6	NW-1	219.8 ^b^ ± 2.3
NR-4	214.8 ^b^ ± 4.7	NW-2	260.2 ^a,d^ ± 3.8
NE-1	278.8 ^c^ ± 2.0	NW-3	301.0 ^e^ ± 3.3
NE-2	267.1 ^d^ ± 2.6	WR-1	229.2 ^b^ ± 0.3

^1^ mg of gallic acid equivalent (GAE)/g extract. ^2^ Values are expressed as mean ± standard deviation (S.D.). ^3^ Different superscript letters indicate differences are significant at *p* < 0.05 using one-way analysis of variance (ANOVA) with a Tukey post hoc as statistical test.

**Table 5:** DPPH antioxidant activity of extracts from *C. longa* rhizomes.

Product	IC_50_ (µg/mL) ^1,2^	Product	IC_50_ (µg/mL) ^1,2^
NR-1	21.22 ^a^ ± 0.19	NE-3	19.04 ^f,g^ ± 0.65
NR-2	23.32 ^b^ ± 0.25	NE-4	18.51 ^h^ ± 0.09
NR-3	28.01 ^c^ ± 0.52	NW-1	25.07 ^f^ ± 0.72
NR-4	29.12 ^d^ ± 0.88	NW-2	19.41 ^b^ ± 0.16
NE-1	16.07 ^e^ ± 0.10	NW-3	15.21 ^g^ ± 0.01
NE-2	19.27 ^f,g^ ± 0.05	WR-1	22.92 ^e^ ± 0.03

^1^ Values are expressed as mean ± standard deviation (S.D.). ^2^ Different superscript letters indicate that differences are significant at *p* < 0.05 using one-way analysis of variance (ANOVA) with a Tukey post hoc as statistical test.

**Table 6:** NO scavenging activity of extracts from *C. longa* rhizomes.

Product	IC_50_ (µg/mL) ^1,2^	Product	IC_50_ (µg/mL) ^1,2^
NR-1	73.9 ^a^ ± 1.4	NE-3	69.0 ^a,b^ ± 4.6
NR-2	78.4 ^a^ ± 5.9	NE-4	65.5 ^a,b^ ± 1.0
NR-3	81.9 ^a^ ± 4.1	NW-1	79.2 ^a^ ± 5.2
NR-4	78.5 ^a^ ± 2.3	NW-2	67.9 ^a,b^ ± 4.0
NE-1	54.3 ^b^ ± 0.4	NW-3	52.5 ^b^ ± 4.1
NE-2	68.1 ^a,b^ ± 3.3	WR-1	69.7 ^a,b^ ± 1.4

^1^ Values are expressed as mean ± standard deviation (S.D.). ^2^ Different superscript letters indicate that IC_50_ differences are significant at *p* < 0.05 using one-way analysis of variance (ANOVA) with a Tukey post hoc as statistical test.

## Data Availability

The data presented in this study are available within this article.
